# Using the deductible for patient channeling: did preferred providers gain patient volume?

**DOI:** 10.1007/s10198-015-0711-z

**Published:** 2015-08-01

**Authors:** Stéphanie A. van der Geest, Marco Varkevisser

**Affiliations:** Institute of Health Policy and Management (iBMG), Erasmus University Rotterdam, P.O. Box 1738, 3000 DR Rotterdam, The Netherlands

**Keywords:** Preferred providers, Patient channeling, Difference-in-difference, I11, I13, C23

## Abstract

In market-based health care systems, channeling patients to designated preferred providers can increase payer’s bargaining clout, other things being equal. In the unique setting of the new Dutch health care system with regulated competition, this paper evaluates the impact of a 1-year natural experiment with patient channeling on providers’ market shares. In 2009 a large regional Dutch health insurer designated preferred providers for two different procedures (cataract surgery and varicose veins treatment) and gave its enrollees a positive financial incentive for choosing them. That is, patients were exempted from paying their deductible when they went to a preferred provider. Using claims data over the period 2007–2009, we apply a difference-in-difference approach to study the impact of this channeling strategy on the allocation of patients across individual providers. Our estimation results show that, in the year of the experiment, preferred providers of varicose veins treatment on average experienced a significant increase in patient volume relative to non-preferred providers. However, for cataract surgery no significant effect is found. Possible explanations for the observed difference between both procedures may be the insurer’s selection of preferred providers and the design of the channeling incentive resulting in different expected financial benefits for both patient groups.

## Introduction

In several countries, deregulation of pricing and the rise of managed care have led to a market-based health care system in which health care providers typically negotiate contracts separately with each third-party payer.^1^ From the perspective of the payer, forming limited or tiered provider networks is a strategic choice to create competition among providers. It may endow the health insurer or other third-party payer with the power to negotiate better deals with providers. The promise of an extra volume of patients may stimulate providers to offer more favorable contract terms (such as price discounts and quality improvements) to the insurer than its competitors do. Sorensen [25] and Wu [28] attempted to empirically measure the effect of ‘moving market share’ to preferred providers on negotiated price discounts. Their findings suggest that health insurers which are better able to channel patients to preferred providers can indeed negotiate better deals with hospitals.

Another health care sector with a similar bargaining setting is the wholesale market for pharmaceuticals. Research by Ellison and Snyder [5] suggest that negotiated discounts in this industry are sensitive to buyers’ abilities to substitute across competing drug products. To influence consumer choice of prescription drugs, health insurers use formularies and financial incentives. For example, patients pay lower or no copayments when they choose drugs that are preferred by their health insurer. Several studies show that these financial incentives are effective at both changing prescribing patterns and moving market share to preferred drugs [8–10, 14].

Other than for prescription drugs, financial incentives are also increasingly used to influence patient choice of health care provider. These incentives include, for example, (i) charging differential copayments across provider tiers, (ii) requiring percentage coinsurance which automatically tiers providers according to price, or (iii) establishing a reimbursement limit which requires the patient to pay the difference between this limit and the insurer-provider negotiated price [15, 16].^2^ Generally speaking, we expect that channeling patients to preferred health care providers is more difficult than for pharmaceuticals, because of typically less observable differences in clinical and non-clinical quality and patients’ distance (travel time) to alternative providers.

To date, the health economics literature provides only limited evidence based on real world data that financial incentives (i.e. cost sharing differences across providers) are effective at encouraging patients to choose preferred providers. Scanlon et al. [20] examined whether waiving standard coinsurance for patients who chose safer hospitals, at a large manufacturing company headquartered in the Midwest of the United States, changed hospital admissions patterns by estimating patients’ probability of choosing a specific hospital. Their findings suggest that the financial incentive significantly influenced patient choice behavior. Rosenthal et al. [19] studied the effect of excluding physicians from a preferred provider organization network in the Las Vegas (Nevada) metropolitan area resulting in higher out-of-pocket payments to see an out-of-network physician. They found that this network narrowing indeed reduced the odds of continuing to see an excluded physician. Robinson and Brown [17] evaluated the impact of an initiative with reference pricing (reimbursement limit) on patient provider choices for orthopedic surgery in California and concluded that it encouraged patients to select low-price facilities. In a more recent study, Robinson et al. [18] examined the effect of another reference-based benefit design in California that financially encouraged patients to select lower-price ambulatory surgery centers for cataract surgery instead of hospital outpatient departments. Their results show that the introduction of this benefit design was associated with a significant increase in patients’ ambulatory surgery center use. Using data from health plans in Massachusetts, Sinaiko and Rosenthal [23] assessed whether tier-rankings had an impact on physician market shares. Overall, they found patients to be quite loyal to physicians. Patients who stayed with their plan year to year were no more likely to switch away from lower-tiered physicians than higher-tiered physicians. The tiering did, however, appear to impact physician market share through the channeling of new patient visits away from the lowest-tiered physicians. Finally, Frank et al. [7] studied a three-tiered hospital network in Massachusetts employing large differential cost sharing to encourage patients to seek care at hospitals in the preferred tier. Their study shows that the tiered network indeed steered patients toward preferred hospitals for planned admissions.

Outside the context of the US health care system, Boonen et al. [2] examined how patients responded to incentives used by two Dutch health insurers to influence the choice of pharmacy. Based on the effects found for two natural experiments, they concluded that patients are sensitive to rather small incentives and that temporary incentives may have a long-term effect on provider choice in the market for outpatient prescription drugs.

Related to the empirical studies discussed above, this paper analyzes a natural 1-year experiment in which a large regional Dutch health insurer designated preferred providers for two different procedures: cataract surgery and varicose veins treatment. Its enrollees were given a positive financial incentive for choosing these providers. That is, patients were exempted from paying their deductible when they went to a preferred provider. Using unique 3-year panel data, we took the providers’ perspective and examined whether preferred providers gained patient volume relative to non-preferred providers caused by patients acting—at least to some extent—as price sensitive consumers of health care.^3^ The paper proceeds as follows. In “Background” the natural experiment is presented in detail. “Data and method” describes both the data and method used for the empirical analysis. In “Results” the estimation results are presented. “Conclusion and discussion” concludes the paper with a discussion of our findings.

## Background

In the new Dutch health care system with regulated competition, introduced in 2006, it is mandatory for all citizens to buy standardized basic health insurance covering the costs of common medical care including primary care, hospital services (for up to 1 year), and pharmaceuticals.^4^ The premium for basic health insurance is community-rated. Every adult has a mandatory annual deductible (155 € in 2009) that must be met (excluding primary care and maternity care) before medical services are reimbursed by the insurer. Consumers obtain a discount on their premium if they opt for a voluntary deductible (at most 500 €). These premium discounts may differ by insurer. In addition to the mandatory deductible and any voluntary deductible, enrollees pay a copayment (a fixed euro amount) for some medical care (e.g. durable medical equipment, certain pharmaceuticals). Overall, from an international perspective, out-of-pocket health care spending in the Netherlands was, in 2009, the lowest of all OECD countries [13].

Competing private health insurers are provided with financial incentives as well as tools to organize and manage acute (curative) care for their enrollees by establishing and maintaining provider networks. Insurers have the legal discretion to engage in selective contracting. That is, they are allowed to form limited provider networks. In 2009, insurers were still very reluctant to limit their provider network for hospital services. Only one very small health plan (13,000 enrollees) provided as of January 2008 access to a limited network of hospitals [11]. An important explanation for this reluctance was that a vast majority of enrollees did not believe that insurers with restrictive networks were committed to provide good quality care [3].^5^

For channeling their enrollees to contracted providers, insurers are legally allowed to use out-of-network cost sharing. A health insurer may require coinsurance to visit a non-contracted provider, discouraging the use of this provider. In addition to selective contracting, insurers are allowed to designate preferred providers within their provider network (i.e. forming tiered provider networks). To encourage patients to visit one of the designated preferred providers, an insurer may decide to waive the annual deductible when they choose to do so. In an attempt to stimulate Dutch health insurers to manage care, they have been permitted by law to apply this positive channeling incentive (i.e. financially rewarding preferred provider choices) since 2009. It provides insurers with another instrument to differentiate cost sharing rates across provider tiers.

In 2009, 15 health insurers, representing about 58 % of all Dutch enrollees, used a differential deductible for channeling patients to preferred providers [12]. One of these insurers was De Friesland Zorgverzekeraar (DFZ), the largest regional health insurer in the Netherlands with a market share of about 65 % in the Dutch province Friesland (or Frisia).^6^ At the national level DFZ had a market share of only 3 % in 2009 [12].

Starting from January 2009, DFZ designated preferred providers for two medical procedures: cataract surgery and varicose veins treatment. For each procedure a set of providers was recognized as preferred because of above average performance on guideline adherence, waiting time and patient satisfaction. Each set included three hospitals and one freestanding ambulatory surgery center. The largest hospital in Friesland was selected for both procedures. In the communication to enrollees it was explained that the preferred providers were carefully selected for reasons of quality. Some positive points for each preferred provider were summed up, such as the fact that a first appointment was possible within 1 or 2 weeks. More detailed information about the selection process was not disclosed. Furthermore, DFZ pointed out that one would be exempted from paying the deductible when visiting a preferred provider. This exemption would concern both the mandatory deductible (155 € in 2009) and, where relevant, the voluntary deductible (at most 500 €).^7^ Since for both procedures the national average of the insurer-provider negotiated prices far exceeds the maximum deductible,^8^ the difference in cost-sharing across the two tiers of providers could add up to a maximum of 655 €. However, the exemption would only apply for cataract surgery or varicose veins treatment. Enrollees still had to pay their annual deductible when using other medical services. With this incentive design, the financial benefit of choosing a preferred provider was different among patient groups. Enrollees who opted for a voluntary deductible in 2009 had a higher potential financial benefit than enrollees with no voluntary deductible. Patients with other medical expenses in 2009 had a small financial benefit or no benefit at all.

In September 2009, DFZ decided to stop using the differential deductible to channel patients as of January 2010 before properly evaluating its effect on the allocation of patients across providers. According to a press release issued by DFZ, the main reason underlying this over-hasty decision was that a majority of enrollees reacted negatively towards the differential deductible. They said that they experienced it as an infringement on their freedom to choose their own provider. Moreover, DFZ admitted that due to a lack of reliable indicators it proved to be very difficult to select providers performing above average on clinical quality. To prevent any negative effects, i.e. losing market share during the open enrollment period in December 2009, DFZ therefore decided rather early to discontinue this financial channeling incentive. Notice that because the experiment did not continue after 1 year, the possibility that patients may have (better) learned about the channeling incentive and its financial benefit in later years was ruled out beforehand.

## Data and method

From DFZ we obtained for both procedures provider claims data for the period January 2007 through December 2009, including the provider name, date of admission and patient’s zip code. In this study we only used claims concerning patients residing in Friesland because the overwhelming majority of this health insurer’s enrollees reside in this province. For all DFZ enrollees who needed treatment for varicose veins and cataract in 2007–2009 as much as 85 and 93 %, respectively, lived in Friesland. Since the upper northwestern part of the country is clearly the key geographical market of this insurer, it is not surprising to find that all preferred providers are situated in the north of the Netherlands. Therefore, we focused our analysis on providers in this part of the country that had a contract with DFZ during each year of the period 2007–2009. For both procedures in the sample period, DFZ did not contract providers selectively. We only included providers which admitted at least one enrollee in each sample year. Annually, these providers accounted for around 98 % of the number of Frisian enrollees needing treatment for varicose veins. For cataract surgery this percentage was even closer to 100 %.

**Fig. 1:**
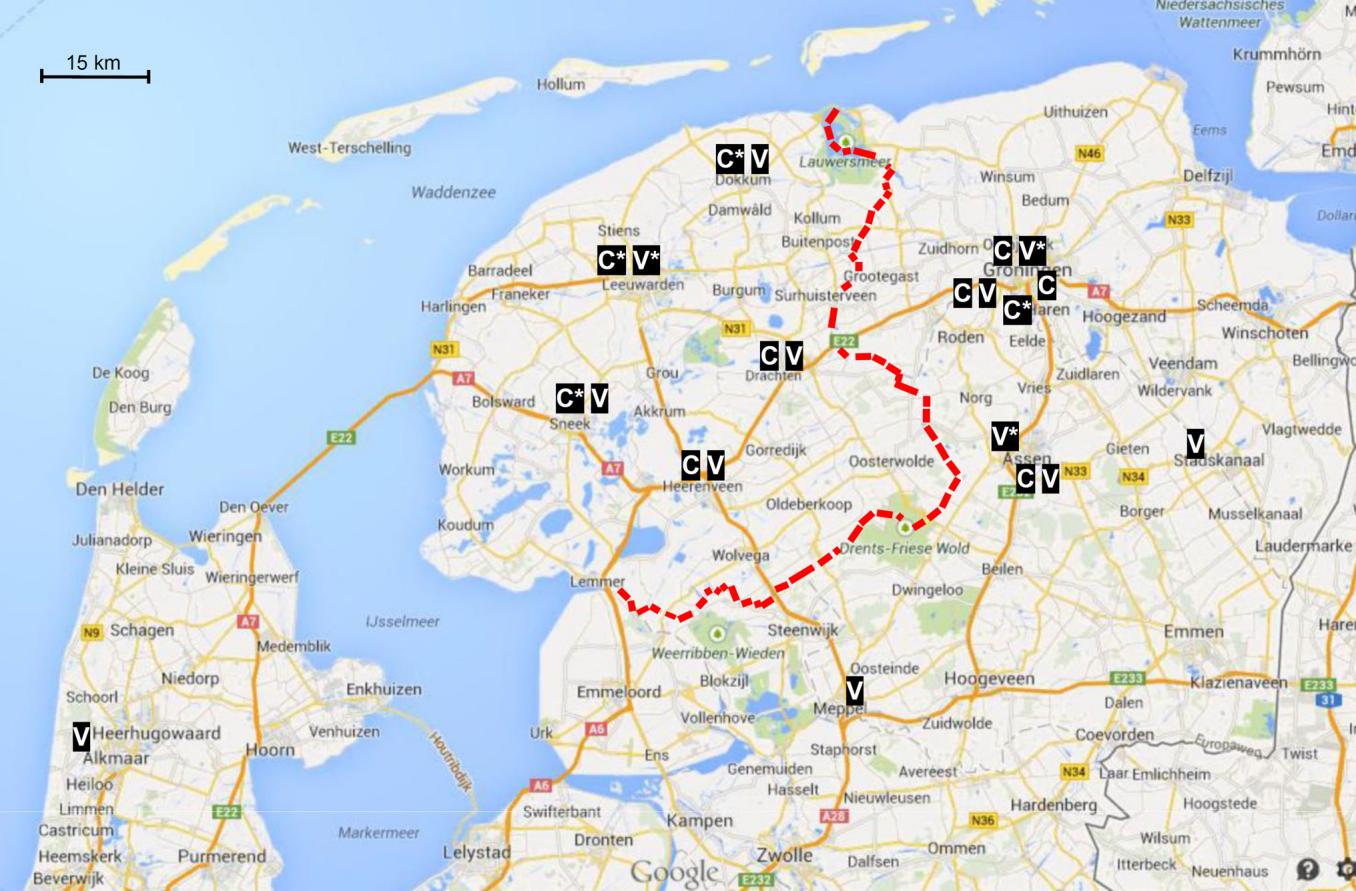
Location of providers included in the two study samples. Providers treating varicose veins and cataract are denoted with V and C, respectively. Preferred providers are marked with an *asterisk*. The province of Friesland is denoted by the *thick dashed line* to the east and by the sea to the west

Our panel of providers delivering cataract surgery to DFZ insured patients contained two ambulatory surgery centers (both in the city of Groningen), one university hospital (also in the city of Groningen) and seven general hospitals. As illustrated in Fig. 1, four of them were designated as preferred provider. For varicose veins treatment, the provider panel included two ambulatory surgery centers (both located outside the Frisian province in the cities of Alkmaar and Assen), one university hospital (in the city of Groningen) and 9 general hospitals. Three of these providers were designated by the insurer as preferred providers.^9^

Each study sample contained data on patient volume for each individual provider for the years 2007, 2008, and 2009. To calculate the total number of patients per provider per year we used each patient’s first visit in the calendar year for that procedure. About one third of the patients in each study sample required more than one treatment.^10^ Because the percentage of these patients choosing different providers was negligible (1.4 and 0.3 % for varicose veins and cataract surgery, respectively), patients were included only once to avoid double counting.

Table 1 provides the descriptive statistics of the variable patient volume for the two study samples. From this Table it follows that for both procedures, variation in the number of patients across providers was substantial during the 3-year study period.

**Table 1:** Patient volume per year, by study sample

	Varicose veins (*n* = 12)			Cataract (*n* = 10)		
	2007	2008	2009	2007	2008	2009
Mean	162	182	186	297	345	300
Std. dev.	216	248	251	312	359	321
Minimum	1	1	1	2	2	2
Maximum	646	760	798	733	817	845

To study the effect of the preferred provider status on the allocation of patients across providers, we used a difference-in-difference approach. Providers in the sample that were not designated as preferred provider in 2009 served as the control group. Table 2 shows for both study samples total patient volume data broken down by preferred provider status and year. As described above, three of the providers in the varicose veins sample were designated preferred provider in 2009 and four providers in the cataract sample. When considering Table 2, the most interesting observation is that in the varicose veins sample the preferred providers in 2009 jointly experienced an increase in patient volume, while their non-preferred competitors suffered a decrease in patient volume. In the cataract sample this difference is not observed. In this market the preferred providers and non-preferred providers both suffered a substantial decrease in number of patients, though the percentage loss of patients was slightly smaller for the first group of providers.

**Table 2:** Patient volume of preferred providers and non-preferred providers, by study sample

	*N*	2007	2008	∆ (%*)*	2009	∆ (%)	Total
Varicose veins							
Preferred providers	3	712	839	+17.8	931	+11.0	2482
Non-preferred providers	9	1235	1343	+8.7	1297	−3.4	3875
Total	12	1947	2182	+12.1	2228	+2.1	6357
Cataract							
Preferred providers	4	2000	2311	+15.6	2016	−12.8	6327
Non-preferred providers	6	968	1142	+18.0	987	−13.6	3097
Total	10	2968	3453	+16.3	3003	−13.0	9424

To test whether the status of preferred provider on average had a statistically significant impact on patient volume, we estimated two regression models: a fixed-effects model and a first-difference model. Since these models are both very useful for program evaluation and one is not better than the other [27], we used them both to see whether or not they give the same results.

In the fixed-effects model, provider fixed effects were included to prevent a bias in the coefficient for preferred provider status resulting from omitted variables. Hence, we used provider fixed effects to remove unobserved variations that were correlated with both preferred provider status and patient volume. The provider fixed effect (*a*_*i*_), or unobserved provider effect, captured all factors affecting patient volume that were generally time-constant in the 3-year study period. In addition to, for example, the provider’s geographical location and its size, these effects also included such attributes as clinical quality and reputation.^11^ Similar to Sivey [24],^12^ we used provider fixed effects to improve the validity of the estimate of the preferred provider status coefficient, which was our only interest. The resulting fixed-effects model for patient volume was:^13^

1 $${\text{Patients}}_{it} = \beta_{1} {\text{PREF}}_{it} + \beta_{2} {\text{d2008}}_{t} + \beta_{3} {\text{d2009}}_{t} + a_{i} + \varepsilon_{it} ,$$

where *i* denotes different providers and *t* denotes year of admission (2007, 2008 or 2009). Hence, the total number of observations is 36 and 30 for the study sample varicose veins and cataract, respectively. The vector *a* includes the provider fixed effects. The variables d2008 and d2009 are dummy variables for 2008 and 2009, respectively. The key independent variable is whether in the year of the experiment a provider was designated as preferred provider or not (PREF). The estimated coefficient *β*_1_ represents the average change in patient volume for the preferred providers compared to the non-preferred providers, other things being equal.

In the first-differenced equation each variable is differenced over time. As a result, the provider fixed effects (*a*_*i*_) drop out. This gives:

2 $$\Delta {\text{Patients}}_{it} = \beta_{1} \Delta {\text{PREF}}_{it} + \beta_{2} \Delta {\text{d2008}}_{t} + \beta_{3} \Delta {\text{d2009}}_{t} + \Delta \varepsilon_{it} ,$$

where *i* again denotes different providers and *t* now refers to either 2008 or 2009. Hence, the total number of observations used for estimating the first-differenced equation is 24 and 20 for the study sample varicose veins and cataract, respectively. As explained above, again our primary interest is in coefficient *β*_1_.

## Results

The top set of Table 3 shows the results of the fixed-effects estimation. The bottom set of results is based on the first-difference equation.

**Table 3:** Fixed-effects and first-difference estimation of patient volume equation

	Varicose veins		Cataract	
	*β*	SE	*β*	SE
Fixed-effects				
PREF	50.94**	20.10	−23.54	31.05
d2008	19.58*	10.05	48.50**	17.57
d2009	10.68	11.24	12.92	21.51
Constant	162.25***	7.11	296.80***	12.42
Obs.	36		30	
* R* ^2^	0.38		0.37	
First-difference				
∆PREF	35.78	21.51	−47.92	37.82
∆d2008	19.58**	9.31	48.50**	18.53
∆d2009	14.47	14.22	22.67	30.25
Obs.	24		20	
* R* ^2^	0.26		0.46	

The intercept reported in the fixed effects estimation is the average of the provider-specific intercepts (*a*_*i*_)

* *p* = 0.1; ** *p* = 0.05; *** *p* = 0.01

The fixed-effects results indicate that the preferred provider status had a significant effect on the allocation of patients across providers treating varicose veins. For this treatment, being designated as preferred provider was, on average, associated with an increase of 51 varicose veins patients per year. The average preferred provider treats about 276 patients per year, so for this hypothetical provider the percentage change in volume was about 18 %. The coefficient on d2008 indicates that total patient volume (i.e. aggregated for all providers) substantially increased from 2007 to 2008.

The estimate in the first-difference equation also suggests that preferred provider status on average increased patient volume, but it is not statistically significant (*p* value = 0.11). This may be the result of the decreased sample size. Again, the intercept for 2008 in this model shows that patient volume increased significantly for all providers in this year. Based on the *R*^2^ it can be concluded that the fixed-effects estimation better explains the observed variation in providers’ patient volume when compared to the first-difference equation.

In contrast to the impact on providers treating varicose veins, the preferred provider status does not seem to increase patient volume for preferred providers of cataract surgery. The coefficient on the preferred provider status variable is in both model specifications not statistically different from zero.

## Conclusion and discussion

Forming preferred provider networks may increase an insurer’s bargaining clout if designating preferred providers has a significant effect on the allocation of patients across individual providers. The results from our analysis, using claims data from a unique natural experiment where enrollees from a large regional Dutch health insurer were exempted from paying their deductible if they went to a preferred provider, suggest that this strategy can be effective in changing the allocation of patients across providers. We found evidence that preferred providers of varicose veins treatment on average experienced a significant increase in patient volume relative to non-preferred providers. However, for cataract surgery no significant effect was found. We can think of two possible reasons for the observed difference in effectiveness between both procedures.

First, in the year prior to the experiment, the joint market share of the preferred providers for varicose veins treatment (38 %) was substantially smaller than the joint market share of the preferred providers for cataract surgery (67 %). Other things being equal, the higher this joint market share, the lower the percentage of patients expected to change from non-preferred to preferred providers in 2009. As a result of the insurer’s selection of preferred providers, the potential number of cataract patients not yet choosing a preferred provider was simply much smaller than for varicose veins patients, which provides an ex ante explanation for the observed difference in the channeling strategy’s effectiveness between both procedures.

Second, the expected financial benefit associated with choosing a preferred provider may have been higher for varicose veins patients than for cataract patients. Due to the design of the channeling incentive, the deductible exemption was only relevant for cataract surgery or varicose veins treatment. According to data provided by the insurer, the group of varicose veins patients was on average much younger than the cataract patient group (51 and 73 years, respectively). Consequently, given that health care expenditures increase with age, we expect varicose veins patients to have, on average, lower expenses for other medical services than cataract patients. Hence, their probability of exceeding the annual deductible was likely to be lower and the expected financial benefit of choosing a preferred provider therefore higher. This potential effect may have been strengthened by the fact that 3 times as many varicose veins patients as cataract patients opted for a voluntary deductible additional to the mandatory one in 2009 (2.1 and 0.7 %, respectively). As a result, there are reasons to assume that the differential deductible was more effective as a channeling instrument for varicose veins patients than for cataract patients.

In summary, our results suggest that the insurer’s patient channeling experiment in 2009 changed the allocation of varicose veins patients across providers. That is, a significant increase in patient volume for preferred providers treating varicose veins was found. However, whether this increase was sufficient to strengthen the bargaining power of the insurer, resulting in lower prices and/or better quality, is an interesting empirical question that unfortunately cannot be answered with the available data. Future research should focus on the extent to which insurers’ channeling strategies motivate health care providers to improve their performance.

## Footnotes

1 These countries include, for example, the Netherlands, Switzerland and the United States.

2 The latter is known as reference pricing and can be interpreted as a reverse deductible. The insurer, rather than the enrollee, pays the first part of the negotiated fee, up to the reimbursement limit, and the enrollee pays the rest.

3 Note that the patient channeling experiment did not increase the insurer’s total patient volume because (i) both procedures were only available to patients after a referral from their general practitioner based on their medical needs, and (ii) patients were legally not allowed to switch health insurer during the year 2009 to have their deductible waived for the procedure.

4 For more detailed information about the Dutch health care system, see for example Schut and Van de Ven [22] and Schut et al. [21].

5 These sentiments are comparable to the managed care backlash that occurred in the United States during the late 1990s [1, 6]. However, the difference is that the Dutch backlash had already occurred before managed care was being applied on a large scale.

6 Friesland is a province in the northwest of the Netherlands. Most of Friesland is on the mainland, but it also includes four small islands which are connected to the mainland by ferry. The total land area equals 3349 km^2^. Friesland has a total population of 646,000 and a population density of 190 inhabitants /km^2^: in 2010 the penultimate population density in the Netherlands.

7 Some other insurers chose to apply the exemption only for the mandatory deductible.

8 This information is obtained from the website http://www.opendisdata.nl/ (accessed on July 17, 2014). DFZ did not provide us with information on their negotiated prices for confidentiality reasons.

9 The fourth provider designated as preferred provider by DFZ, a general hospital located relatively far away, was not included in our study because none of the Frisian enrollees of DFZ visited this provider in the sample period.

10 If there are cataracts in both eyes that require surgery, the surgeries are normally not performed at the same time. Also, for people who have varicose veins in both legs, treatment is commonly performed apart.

11 At the individual provider level, overall reputation (as measured by a popular Dutch news magazine) was indeed strongly correlated over the 3-year time period. The availability of public information about clinical quality was very limited in the sample period.

12 In his study, Sivey [24] examined patient hospital choice for cataract surgery in the United Kingdom, concentrating on the trade-off between travel time and waiting time.

13 This model is similar to the equation used by Cutler et al. [4] examining whether quality report cards in New York State and Pennsylvania affected the distribution of patients across individual providers of bypass surgery. Wang et al. [26] adopted a similar estimation strategy when examining the impact of report cards on providers’ patient volume in the market for bypass surgery in Pennsylvania.
